# Visuospatial working memory and the construction of a spatial situation model in listening comprehension: An examination using a spatial tapping task

**DOI:** 10.1007/s10339-021-01063-0

**Published:** 2021-11-09

**Authors:** Yun Lin, Norio Matsumi

**Affiliations:** Graduate School of Education, Hiroshima University, Hiroshima, Japan

**Keywords:** Listening comprehension, Spatial situation model, Visuospatial working memory, Verbal working memory, Spatial tapping task

## Abstract

The present study investigated how visuospatial working memory (VSWM) is involved in the construction of a spatial situation model for spatial passages presented auditorily. A simple spatial tapping condition, a complex tapping condition as a target-tracking task, and a control condition, were used to analyze the role of VSWM. To understand how individuals who differ in verbal working memory (VWM) capacity (determined with a listening span test) process spatial text during dual-task performance, individual differences in VWM capacity were analyzed. In two experiments, the participants listened to a spatial text at the same time as performing a spatial concurrent task or no concurrent task. The results of the free recall test in Experiment 1 showed that there were no differences between the tapping conditions in the high VWM capacity group; the low VWM capacity group had a lower performance in both spatial tapping tasks compared to the control condition. The results of the map drawing test in Experiment 2 showed that complex spatial tapping impaired performance in comparison to simple spatial tapping and the control condition in the high VWM capacity group; in the low VWM capacity group, both spatial tapping tasks impaired recall performance. In addition, the participants with high VWM capacity demonstrated better performance. Overall, the results suggest that individuals with high VWM capacity have more resources to process verbal and spatial information than those with low VWM capacity, indicating that VWM capacity is related to the degree of the involvement of VSWM.

## Introduction

In the processing of text comprehension, three levels of representation are constructed (van Dijk and Kintsch 1983). At the lowest level is the verbatim, which is a representation of the surface form of the words and syntax used in the text. At the next highest level is the propositional text-base, which is a representation of the meaning expressed in the text. At the highest level is the situation model, which is a representation of the situation to which the text refers and is dependent on the former representations and the world knowledge of the reader or listener. There is general agreement in the field that the situation model is multidimensional and involves various perceptual components, such as verbal, visual, and spatial representations, depending on the information included in the text or the task requirements (e.g., Friedman and Miyake 2000; Zwaan and Radvansky 1998). For example, for a text involving spatial descriptions, a spatial situation model that represents spatial positional relationships is built (e.g., Bryant et al. 1992; Bower and Morrow 1990; Glenberg et al. 1987; Mani and Johnson-Laird 1982; Morrow et al. 1989; Perrig and Kintsch 1985; Taylor and Tversky 1992). Furthermore, the spatial situation model involves verbal and visuospatial imagery representations (e.g., Jahn 2004; Picucci et al. 2013).

However, a spatial situation model derived from a text is not always fully constructed, since the representations of each sentence are spatially connected to the next sentence during comprehension processing, which increases cognitive load. When a text is presented auditorily, the construction of the spatial situation model is considered to be more difficult since a series of time-based processing steps are required (Anderson 1983). As Anderson (1983) proposed, listening comprehension consists of perception, parsing, and utilization. It is suggested that the verbatim, propositional text-base, and situation model are constructed at each processing level. On the basis of the construction of the spatial situation model in listening comprehension, in which the integration of representations in a variety of modalities are required along the time axis, it is proposed that the process is related to the individual differences of listeners, especially working memory (WM) capacity.

WM is regarded as a temporary processing and storage cognitive system with a limited capacity. The WM model, proposed by Baddeley (2010), consists of three sub-systems, i.e., the phonological loop, visuospatial sketch pad, and episodic buffer, and the main system, i.e., the central executive. The phonological loop holds verbal and acoustic information using a temporary store, called the phonological store, and an articulatory rehearsal system. The visuospatial sketch pad is holds visuospatial information, with a temporary passive store, known as the visual cache, and an active rehearsal system, named the inner scribe. The episodic buffer stores information as a multi-dimensional code, providing a temporary interface between the other two sub-systems and long-term memory (LTM). These sub-systems are controlled by the central executive. WM is further divided into verbal WM (VWM), which combines the functions of the phonological loop and central executive, and visuospatial working memory (VSWM), which combines the functions of the visuospatial sketch pad and central executive (e.g., Kaneda and Osaka 2007). VWM is responsible for language processing, while VSWM maintain visuospatial information and possibly the formation and manipulation of mental images (Baddeley and Logie 1999).

A number of studies using the dual-task paradigm have claimed that VWM and VSWM are involved in the construction of spatial situation models in text processing (e.g., Brunyé and Taylor 2008; De Beni et al. 2005; Pazzaglia et al. 2010; Picucci et al. 2013). In the dual-task paradigm, subjects perform a primary task concurrently with a secondary task. If the primary and secondary tasks share the same WM resource, then performing both tasks concurrently will have a deleterious effect on the performance of one or both tasks (Rende et al. 2002).

De Beni et al. (2005), investigated the involvement of VWM and VSWM in processing spatial and nonspatial texts using a dual-task paradigm. Italian L1 speakers listened to a spatial or nonspatial text, with an articulatory suppression or spatial tapping task as a concurrent task. The results of a free recall test showed that the processing of both texts was influenced differently by both concurrent tasks compared to the control condition, with a disruptive effect by the verbal task on both texts and selective interference by the spatial task on the spatial text. These results confirmed that VWM is involved in the processing of text comprehension and memory, and when the text contains visuospatial information, VSWM also plays an important role in processing the visuospatial components.

Pazzaglia et al. (2010) used similar procedures as De Beni et al. (2005), but with a map drawing test instead of a free recall test, to investigate the involvement of VWM and VSWM in the processing of spatial texts. Their results showed that for spatial text of a route perspective, verbal and spatial interference both impaired performances compared to the control condition, indicating that VWM is involved in language processing itself, whereas VSWM is involved in the development of spatial situation models.

From these findings, it is tempting to conclude that VWM has a role in language processing and VSWM builds visuospatial imagery representations during the construction of the spatial situation model. However, as mentioned above, WM capacity is limited. Whether or not the capacities of VWM and VSWM affect the processing for the construction of the spatial situation model remains to be examined.

VWM capacity, as measured by the Reading Span Test or Listening Span Test (LST) developed by Daneman and Carpenter (1980), is considered to be a predictor of listening comprehension ability (Daneman and Merikle 1996). Saito and Miyake (2000) noted that these tests reflect the efficiency of language processing. Daneman and Carpenter (1980) pointed out that readers or listeners with high VWM capacity might benefit from their efficiency at chunking in comprehension and WM span tasks. Nishizaki and Osaka (2001) indicated that listening comprehension includes two types of processing, i.e., phonological, which consists of the temporary retention of phonological information and the retrieval of phonological representations in LTM as needed, and semantic, which consists of the retention of information as higher-level semantic content and the retrieval of semantic representations in LTM as needed. Therefore, the difference in listening comprehension due to VWM capacity depends on the efficiency of the connection between phonological and semantic processing. Applying the above to the model of Anderson's (1983), listeners with high VWM capacity should be more efficient at integrating phonological and semantic information in the episodic buffer and constructing propositional representations by the intervention of the central executive when parsing than those with low VWM capacity.

Conversely, VSWM capacity, as commonly measured by forward and backward spatial span tasks, also known as the Corsi block-tapping task (Corsi 1972), reportedly predicts participants' performance in spatial thinking (e.g., Friedman and Miyake 2000). Several studies have found that individuals with high spatial span, evaluated using Corsi block-tapping task, have better spatial text recall than those with low spatial span (e.g., Gyselinck et al. 2007; Pazzaglia and Cornoldi 1999). Mediation model studies have also shown that VSWM capacity, measured with the backward version of the Corsi block-tapping task, directly affects spatial text recall, and at the same time mediates the relationship between spatial ability and the spatial recall test (e.g., Meneghetti et al. 2011, 2014).

Therefore, it is conceivable that the construction of the spatial situation model in listening comprehension, which is considered to consist of two temporally related processes, i.e., construction of the propositional text-base through language processing and construction of the situation model through the constructed propositional text-base (e.g., Johnson-Laird 1983; Perrig and Kintsch 1985; Glenberg et al. 1994), is related to a listener’s VWM and VSWM capacity.

When a spatial text is presented verbally, the following subprocesses can be assumed: perceptive analysis of words in the input sentence; formation and storage of verbal representations; activation of imagery representations; and integration of the verbal and imagery representations to build a spatial situation model. The spatial situation model derived from auditorily presented texts is built incrementally by the integration of successive verbal inputs and imagery in the course of listening.

**Fig. 1:**
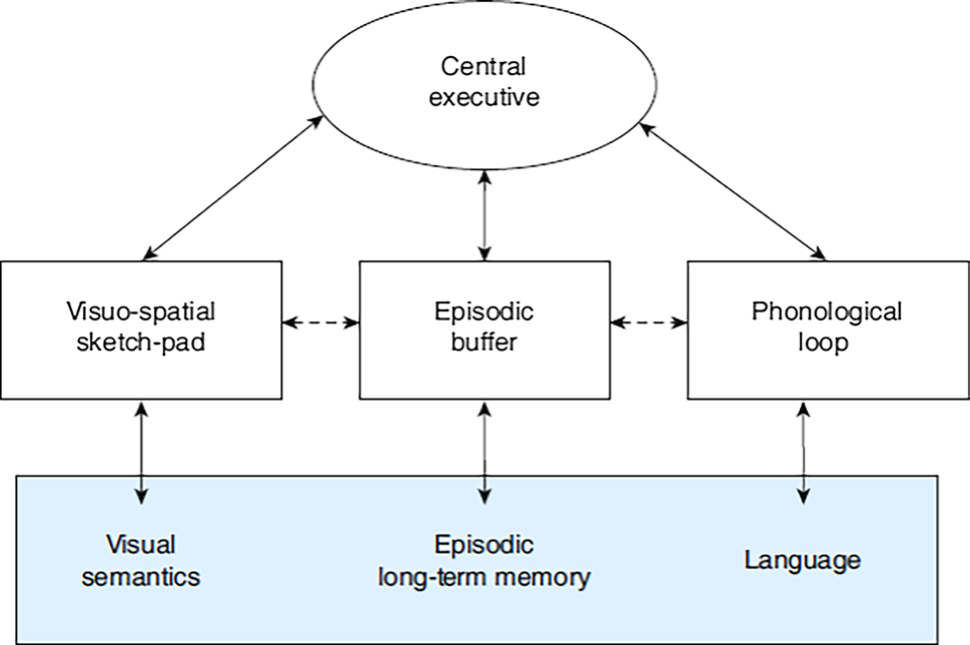
A later development of the multicomponent model (Baddeley 2010)

In line with the WM model (Baddeley 2010; Fig. 1), the functions of the processing subsystems for constructing the spatial situation model are assumed as follows. Verbal input firstly enters the phonological loop, where it is retained temporarily through articulatory rehearsal, and the corresponding linguistic meanings are activated through interactions with language-related LTM. Then, the verbal representations are constructed in the episodic buffer. During their temporary retention in the episodic buffer, the corresponding visuospatial images are activated through interactions of the visuospatial pad and visual semantics, and the formed imagery representations are retained in the visual cache through rehearsal in the inner scribe. After temporary storage in the visual cache, the imagery representations are transferred to the episodic buffer. With the continuous input of linguistic information, the imagery representations just transferred to the LTM, while highly activated, bind with the verbal representations constructed along the time axis, and the spatial situation model is updated in the episodic buffer (Fig. 1).

In such cognitive processing, the degree to which VSWM is involved in the construction of the spatial situation model, i.e., the abundance of modalities of the representations constituting the spatial situation model, is assumed to be related to VWM and VSWM capacity.

Several studies have discussed the relationship between VWM and VSWM capacity and context comprehension with correlational analyses (e.g., Friedman and Miyake 2000; Sasaki 2009), and indicated that VWM and VSWM play independent roles in the construction of situation models, consistent with previous studies (e.g., De Beni et al. 2005; Pazzaglia et al. 2010). However, only with the dual-task paradigm or correlational analyses, the question of the parallel work of VWM and VSWM and the effects of limits in capacity in the construction of a spatial situation model seemingly remain unresolvable. During the processing of spatial descriptions in listening comprehension, the processing resources that can be assigned to the phonological loop and visuospatial sketchpad are controlled by the central executive to process verbal and imagery information in parallel. In other words, there should be a competition for processing resources between the two subsystems when both verbal and imagery information are required to process. Therefore, it is estimated that VWM and VSWM capacities measured in a single situation, i.e., with the LST or Corsi block-tapping task, are not likely to be completely reflected in a listener's performance during listening comprehension.

To bridge the gap between how VWM and VSWM work together to process spatial text, we developed a method using a dual-task paradigm with individual differences in VWM capacity, referring to Meneghetti et al. (2009).

Meneghetti et al. (2009) investigated the extent to which VSWM and VWM are involved in spatial text processing when individuals have good or poor mental rotation ability using a dual-task paradigm, i.e., asking good and poor mental rotators to listen to spatial descriptions (primary task) and to perform spatial and verbal secondary tasks loading VSWM and VWM, respectively. They found that the spatial text recall of the low mental rotation group was impaired by verbal and spatial concurrent tasks relative to the control condition, while the spatial text recall of the high mental rotation group was not impaired by either concurrent task. These results confirm the involvement of the VWM and VSWM systems in spatial text processing and suggest that individuals with low mental rotation ability require more verbal resources to process spatial information described verbally.

A mediation model examining how mental rotation, VSWM, and VWM work together to influence the recall of spatial descriptions tested by Meneghetti et al. (2011) also indicated that mental rotation performance, which is related to spatial text recall, is mediated by the active spatial processing of WM and requires a certain level of central executive control, which is consistent with the results of Meneghetti et al. (2009). However, the mediation model also claimed that VWM capacity, measured by the digit span test, was not related to spatial text recall. Some arguments on this point could be raised because the digit span test should be considered as an assessment of verbal short-term memory span (Richardson 2007) rather than VWM capacity. During the processing of spatial texts, verbal processing through VWM and spatial processing through VSWM both adjust the central executive; therefore, to explore the influence of VWM, a more complex test than the digit span test to measure VWM capacity, such as the LST, would be more appropriate.

On the basis of the processing of spatial text, linguistic processing should be considered as a premise for the construction of the spatial situation model. Besides, several studies have confirmed the positive influence of VWM capacity on language comprehension.

In light of the above, to investigate the relationship between VWM capacity and the involvement of VSWM in the construction of the spatial situation model, we analyzed the individual difference in VWM capacity, as measured by the LST. We utilized two tapping conditions that differed in the degree of involvement of the central executive and also a no-tapping control condition to manipulate the available VSWM resources during listening comprehension.

The simple tapping task, which is often used to explore the functions of the inner scribe (e.g., Gyselinck et al. 2002; De Beni et al. 2005), requires continuous tapping on the four corners of a square. Continuous spatial tapping competes with the processing resources of the inner scribe and consequently impairs the maintenance of constructed spatial images in VSWM (Suto 2005). The complex tapping task, set as a target-tracking task, requires continuous tapping of a colored target squares presented randomly on a computer screen. The square appears in a different color each time it is presented. The integration of motor function and visual perception required in the complex tapping task involves more attention control through the central executive than only the motor function required in the simple tapping task. Therefore, complex tapping impairs the formation and manipulation of mental images (Nishizaki 1998) or further causes a redistribution of processing resources in listening comprehension.

In terms of the modalities of the representations involved in the spatial situation model, tests requiring the productions of different representations lead listeners to distribute more processing resources to retain the corresponding representations, leading to a different degree of involvement of VWM and VSWM. Accordingly, we utilized a free recall test for verbal memory (Experiment 1) and a map drawing test for imagery memory (Experiment 2), and the spatial tapping tasks were predicted to have different interference effects in each test.

### Experiment 1

In Experiment 1, we investigated whether the degree of involvement of VSWM depends on VWM capacity when the verbal memory of spatial descriptions is required. The materials used in this study consisted of a protagonist's movements and relevant landmarks, which leads the semantic representations of each sentence to be connected spatially to the next sentence temporally along the time axis. Therefore, to recall a complete verbal memory, the production of bound verbal and imagery representations in the episodic buffer is expected. The reconstruction of linguistic information requires the retrieval of the processed sentences in their input order, which needs additional processing resources to retain the verbal representations. On the basis of the typical interference effect of the tapping tasks observed in the processing of spatial texts found in the general population (De Beni et al. 2005; Gyselinck et al. 2007), interference effects due to the spatial tapping tasks are expected to be observed in both groups. The high VWM capacity group can construct the verbal representations more effectively during text processing and compensate for the interference effects of the simple tapping tasks, which consume a smaller amount of processing resources in VSWM; besides, interference effects are expected to be observed in the complex tapping condition, due to the requirement for more processing resources in VSWM and may impair the formation of imagery representations. Conversely, in the low VWM capacity group, which requires more resources to process linguistic information, interference effects by both concurrent tasks, through which the reallocation of processing resources may occur, are likely to be observed. Correspondingly, a higher recall rate should be observed in the high VWM group than in the low VWM group.

## Method

### Participants

Statistical power analysis was performed for sample size estimation. Effect size was set to 0.25, considered to be a medium level using the criteria of Cohen (1992). With an alpha of 0.05 and power of 0.80, the required sample size was approximately 28 for analysis of variance (ANOVA) with repeated measures for between-within participants interactions (G*Power 3.1; Faul et al. 2007).

A total of 40 undergraduates and graduates (Chinese L1 speakers, 32 females) from Hiroshima University participated in Experiment 1. Their mean age was 25.2 years (standard deviation [*SD*] = 3.02). There were four males and 16 females in both groups. All of the participants were paid 500 yen as a reward.

### Materials

#### Texts

According to studies showing strong VSWM involvement in route perspective descriptions (Brunyé and Taylor 2008; Pazzaglia et al. 2010), three spatial texts describing different routes were constructed. The texts were selected from the listening comprehension questions of the Japanese-Language Proficiency Test, level 1 (N1), and edited by the authors. The Japanese texts were translated into Chinese and checked for grammar, vocabulary, and fluency by two high school teachers of Chinese literature who are Chinese L1 speakers.

Each text contained five landmarks, three descriptions about the protagonist’s movements such as “turn left” and “walk straight” and three descriptions about relative location among the landmarks such as “on the left” and “on the right” (see excerpts in Table 1 and full texts in Appendix 1). All texts were recorded by a Chinese L1 speaker (female) in standard Mandarin at a normal speed. The three texts were of similar length and contained a mean number of 103 words (*SD* = 6.07), and the mean length of the listening materials was 30 s (*SD* = 6.07).

**Table 1:** Excerpts (English translation) from the texts

Text 1	Xiaohua is taking a walk in the square. After entering the square, she walks straight for a little while and sees a fountain on her left…
Text 2	Xiaoming is heading from Central Station to an interview with a company. Donghua Tower is in front of him after leaving the north exit of the station…
Text 3	Xiaodong is participating in a marathon. The starting point of the course was in front of the university…

A pilot study, with six Chinese L1 speakers who did not take part in Experiment 1, was conducted to assess the equivalency of difficulty and confirm the construction of a connected and integrated spatial mental model derived from the three texts. In the pilot study, the participants were instructed to write down the spatial descriptions after listening to the texts. The mean recall rate of the three texts was 70.27 (*SD* = 8.27), and the lack of a significant difference with one-way ANOVA confirmed a comparable completion level of the spatial situation model (*F*(2,17) = 0.02, *p* = 0.981). After the free recall task, a survey on the difficulty of the texts with a five-point evaluation method was also completed by the participants, with one point indicating “extremely easy” and five points indicating “extremely difficult.” The mean score of the three texts was 3.56 (*SD* = 0.51), and the lack of a significant difference with one-way ANOVA also confirmed a comparable level of difficulty (*F*(2,17) = 1.43, *p* = 0.869).

#### LST

We used the Chinese version of the LST, which was an improved version of the one devised by Daneman and Carpenter (1980) to measure VWM capacity. The LST consisted of two to five sentence conditions, each with three sets (see examples in Table 2), and tones were inserted to mark the beginning and end of each set. The Chinese sentences were selected from Yan (2014).

**Table 2:** Examples of sentences in the Listening Span Test

Swimming is a good exercise for the body	⚪
Coffee is a traditional Chinese drink	×

The participants were told to listen to multiple unrelated sentences presented by set and memorize the last word of each sentence while making true/false judgments about the content of the sentence. The true/false judgments while listening to each sentence and word recall after each set ended were required. The participants were given two practice items at the two-sentence condition before the test began.

#### Dual -task

The simple tapping task required the participants to tap the four corners of a 10 × 10 cm square continuously with the dominant hand in a counterclockwise direction at their own pace.

The complex tapping task, which was set as a target-tracking task, required continuous tapping of a single-colored target square presented in a random location on a computer screen, which was divided equally into nine parts. As soon as the participant clicked the presented square correctly, the next square was presented randomly in any of the nine parts of the screen with another single color. The number of correct taps was recorded automatically by the computer.

#### Design

The design was mixed, with VWM capacity (high, low) as a between-participants factor, and the requirements of the three concurrent task conditions (simple spatial tapping task, complex spatial tapping task, and control condition) as a within-participant factor.

#### Procedure

Each participant was tested individually for approximately 30 min. The primary task consisted of the participants listening to the texts only once, with the expectation that they should later write down the descriptions on a sheet of paper. The participants were told before the listening task to imagine they were walking in a city as the protagonist when memorizing the descriptions, based on studies showing that imagery instructions help participants to form a spatial situation model (e.g., Gyselinck et al. 2007, 2009; Meneghetti et al. 2013).

Each text was associated with a different concurrent task condition in a counterbalanced order.

## Results

### LST

The score for the LST was based on true/false judgment and word recall scores, following Daneman and Carpenter (1980), who claimed that the verification component is included in the span measure to ensure that the participants process the entire sentence and do not concentrate only on the final word of each sentence. If two or more of the three sets were answered correctly in the true/false judgment and word recall, it was considered that the corresponding sentence condition had been completed, and one point was given and the next sentence condition was scored. If the number of correctly answered sets was less than 1, it was considered that the corresponding sentence condition had not been completed, and no point was given, and scoring ended. If only one set was answered correctly, 0.5 points were given, and scoring ended.

The initial score was 1.0 and the total score was set to 5.0. The mean score of the 40 participants was 3.88. Twenty participants with a score ≥ 4.00 were placed in the high VWM capacity group (*M* = 4.50, *SD* = 0.40), and the other 20 participants with a score ≤ 3.50 were placed in the low VWM capacity group (*M* = 3.25, *SD* = 0.26). Student’s *t*-test showed a significant difference between both groups (*t*(1, 38) = 6.88, *p* < 0.001, *r* = 0.74). Both groups had the same mean scores in the Corsi block-tapping task (high VWM capacity group: *M* = 6.50, *SD* = 0.69; low VWM capacity group: *M* = 6.50, *SD* = 0.76).

### Dual -task

In the simple task condition, the completion of the task by the participants was confirmed by the experimenter. In the complex task condition, the mean number of correct taps in each text was calculated. Comparisons between the means of the two VWM capacity groups by Student’s *t*-test indicated that there was no difference in the degree of interference due to the complex tapping task between both groups during the listening task (*t*(38) < 0.1, *p* = 0.486, *r* = 0.01).

### Free recall^1^

The score of the free recall test was based on the idea unit (IU; Muramoto 1992). When one single IU was completely recalled, two points were given, for example when the participants wrote a precise description, e.g., “After entering the square, she walks straight for a little while and sees a fountain on her left.” When one single IU was partially recalled, one point was given. For example, when a participant named a landmark (e.g., “a fountain”), but failed to give the location (e.g., “on the left”), or if the location was only partially correct (e.g., “on the right of the square”). When the recalled information was considered inaccurate or incorrect, no score was assigned (e.g., “After entering the square, she walks straight for a little while and sees a bookstore on her right”).

The scores were determined by two independent judges. According to the high consistency between the scores of both judges as determined with Pearson's correlation coefficient (*r* = 0.89, *p* < 0.001), the analyses were carried out on the scores given by the first judge (the experimenter).

**Fig. 2:**
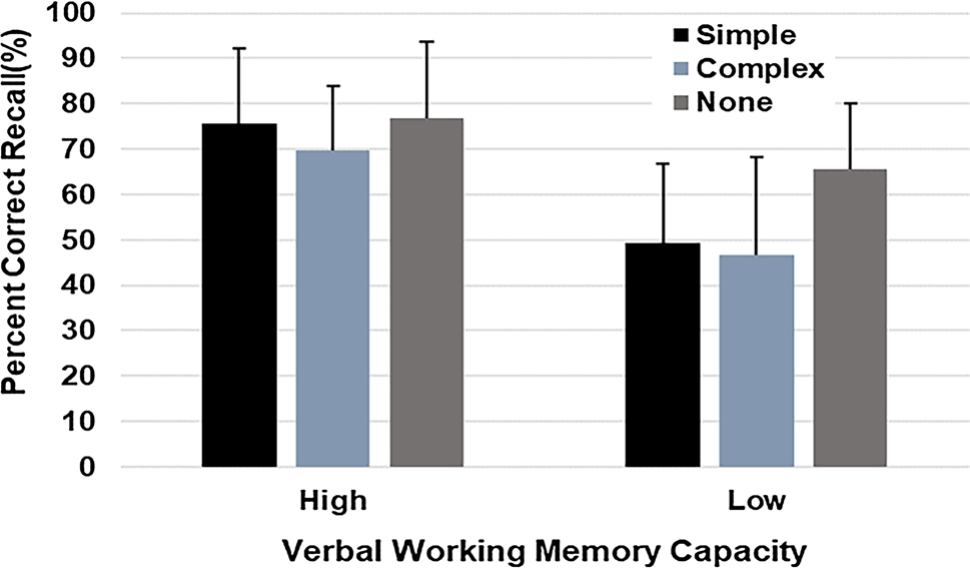
Experiment 1: Significant main effect of verbal working memory capacity, concurrent task, and interaction effect of verbal working memory capacity and concurrent task on the recall rates for the free recall test

Figure 2 shows the mean overall recall rates for the three concurrent tasks. A mixed 2 × 3 ANOVA with VWM capacity as a between-participants factor and the concurrent task as a within-participants factor was carried out. ANOVA yielded a significant main effect of VWM capacity, (*F*(1, 38) = 17.11, *p* < 0.001, η^2^ = 0.31; in this study, partial η^2^ is reflected as η^2^), indicating a higher recall rate in the high VWM capacity group (*M* = 74.04, *SD* = 16.35) compared to the low VWM capacity group (*M* = 53.85, *SD* = 20.13). The main effect of concurrent task condition was also significant; (*F*(2, 76) = 16.95, *p* < 0.001, η^2^ = 0.31). A comparison among pairs of means, using Bonferroni’s method, confirmed that the recall rate was higher in the control condition than in the simple and complex tapping conditions, which showed no difference between each other (control condition, *M* = 71.17, *SD* = 25.67; simple tapping condition, *M* = 62.44, *SD* = 27.63; complex tapping condition, *M* = 58.23, *SD* = 29.63). A significant interaction effect was observed (*F*(2, 76) = 9.10, *p* = 0.001, η^2^ = 0.33). Multiple comparisons with Bonferroni’s method showed no difference in the main effect of concurrent task conditions in the high VWM capacity group (*p* = 0.076), indicating that the recall rate was not significantly different among the three conditions (control condition, *M* = 76.79, *SD* = 17.33; simple tapping condition, *M* = 75.50, *SD* = 17.08; complex tapping condition, *M* = 69.83, *SD* = 14.46). In addition, a significant difference was observed in the low VWM capacity group (*p* < 0.001), confirming that the recall rate was higher in the control condition (*M* = 65.54, *SD* = 15.06) than in the simple (*M* = 49.38, *SD* = 17.86) and complex (*M* = 46.63, *SD* = 22.20) tapping conditions, which were not significantly different between each other.

## Discussion

The results showed that the high and low VWM capacity groups were influenced differently by the two tapping tasks, i.e., with no significant difference between the tasks in the high VWM capacity group, while significantly higher recall rates were observed in the control condition than in both tapping conditions in the low VWM capacity group, which are partially in accordance with our hypothesis. Combined with the observed main effect of VWM capacity, it was shown that verbal and imagery representations are constructed in parallel to a certain degree in both groups, but the degree of imagery representation formation, i.e., the degree of involvement of VSWM was affected by VWM capacity when verbal memory for spatial descriptions was required.

To recall a listened description, it is assumed that a certain amount of processing resources are assigned to retain verbal representations. The lack of a significant difference among the concurrent tasks in the high VWM capacity group suggests that an increased ability to construct verbal representations allows more resources to be distributed to the processing of imagery representations in parallel. Conversely, in the low VWM capacity group, the decreased recall rate during the concurrent tapping tasks indicates that the required body movements deplete the processing resources available for the construction of imagery representations in VSWM. Since a certain amount of resources must be distributed preferentially to the processing and retention of sequential language information input, interference with the generation of imagery representations and further bindings in the episodic buffer occur. This results in the maintenance of more complete bound verbal and imagery representations in the control condition, but only verbal representations and incomplete imagery representations are maintained in the simple and complex tapping conditions.

From the results of Experiment 1, it is inferred that in the same time frame, listeners with high VWM capacity construct more complete verbal representations, allowing more efficient binding with imagery representations than individuals with low VWM capacity when verbal memory is recalled.

### Experiment 2

In Experiment 2, we investigated whether the degree of involvement of VSWM depends on VWM capacity when imagery memory for spatial descriptions is required. The recall of linguistic information as a map requires the retrieval of formed imagery representations transferred from verbal representations. Therefore, participants tend to retain imagery representations rather than verbal representations in the map drawing test. In other words, the bound representations updated along the time axis in the episodic buffer are ultimately retained and produced in the form of imagery representations. Since verbal representations do not need to be retained, which leads to the reduction of required VWM processing resources, it is plausible that more resources could be distributed to VSWM for the construction and retention of imagery representations. Therefore, the high and low VWM capacity groups should be able to construct imagery representations through VSWM in the control condition, and correspondingly, interference due to the spatial tapping tasks is likely to be observed in both groups in the tapping conditions, but the degree of interference is not expected to differ clearly between both groups. In addition, from the point of view that the formation of imagery representations is temporally based on linguistic processing during the construction of a spatial situation model, better performance should be observed in the high VWM group compared to the low VWM group.

## Method

### Participants

A total of 40 undergraduates and graduates (Chinese L1 speakers, 34 females) from Hiroshima University, who did not take part in Experiment 1, participated in Experiment 2. Their mean age was 25.5 years (*SD* = 4.23). There were three males and 17 females in both groups. All of the participants were paid 500 yen as a reward.

### Materials

The texts and listening materials are the same as in Experiment 1. A pilot study, with six Chinese L1 speakers who did not take part in Experiment 2, is conducted as in Experiment 1. In the pilot study, the participants were told to draw a map after listening to the texts. The mean recall rate of the three texts was 76.04 (*SD* = 15.50), and one-way ANOVA confirmed a comparable completion level of the spatial situation model (*F*(2,17) = 0.03, *p* = 0.975). After the map drawing task, a survey on the difficulty of the texts with a five-point evaluation method was conducted. The mean score of the three texts was 3.44 (*SD* = 0.51), and one-way ANOVA confirmed a comparable level of difficulty (*F*(2,17) = 0.14, *p* = 0.869).

### Design

The design is the same as in Experiment 1.

### Procedure

Except that a map drawing test was used instead of the free recall test, and the participants were told before the listening task that they would later draw the described environment as a map on a sheet of paper, the procedure is the same as in Experiment 1.

## Results

### LST

The mean score was 3.84. Twenty participants with a score ≥ 4.00 were placed in the high VWM capacity group (*M* = 4.53, *SD* = 0.44), and the other 20 participants with a score ≤ 3.50 were placed in the low VWM capacity group (*M* = 3.15, *SD* = 0.61). Student’s *t*-test showed a significant difference between both groups (*t*(1, 38) = 6.02, *p* < 0.001, *r* = 0.70). The two groups had the same mean scores in the Corsi block-tapping task (high VWM capacity group: *M* = 6.45, *SD* = 0.83; low VWM capacity group: *M* = 6.40, *SD* = 0.75).

### Dual -task

As in Experiment 1, in the simple task condition, the participants were confirmed to have completed the task by the experimenter. In the complex tapping task condition, comparisons between the mean number of the correct tap between the two VWM capacity groups by Student’s *t*-test did not reveal a significant difference in the degree of interference due to the complex tapping task between the both groups during the listening task (*t*(38) < 0.1, *p* = 0.457, *r* = 0.01).

### Map drawing

Map drawing scoring evaluated landmark recall and relative landmark locations, which assesses the correspondence with landmark-to-landmark comparisons derived from the texts. If the landmark and relative locations were recalled correctly, two points were given, i.e., the precise locations of the landmarks were recalled and the correct relationship with other nearby landmarks was maintained, for example, when the participants put the cafe on the right side of the square with a fountain on the left side. One point was assigned when the participants partially recalled the landmark positions within the environment, for example, when the participants put the cafe on the left side of the square with a fountain on the right side. One point was also assigned when the relative locations were described correctly but without the correct names of the landmarks, for example, when the participants put a blank instead of “cafe” or with other landmarks on the left side of the square with a fountain on the right side.

The scores were determined by two independent judges. According to the high consistency between the scores of both judges as determined with Pearson's correlation coefficient (*r* = 0.90, *p* < 0.001), the analyses were carried out on the scores assigned by the first judge (the experimenter).

**Fig. 3:**
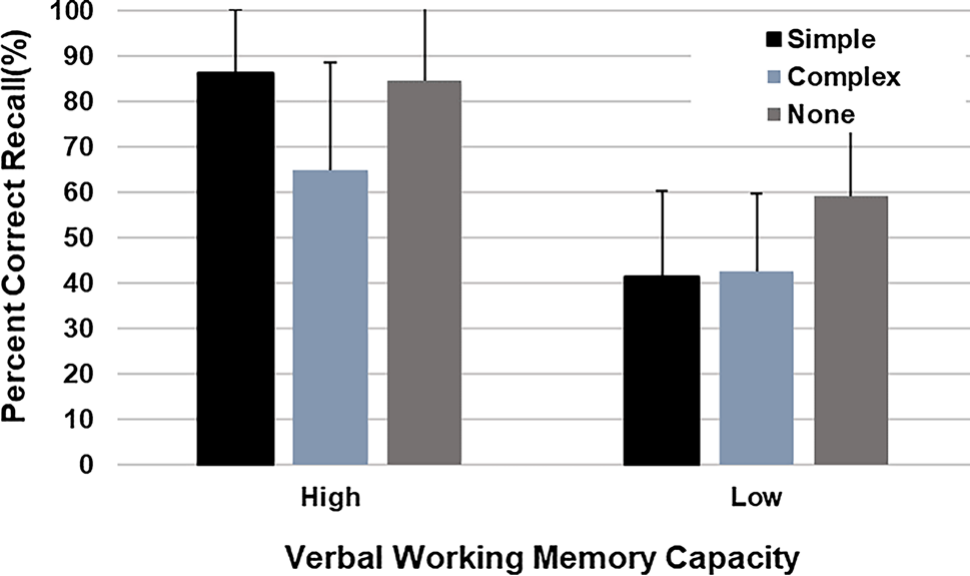
Experiment 2: Significant main effect of verbal working memory capacity, concurrent task, and interaction effect of verbal working memory capacity and concurrent task on the recall rates for the map drawing test

Figure 3 shows the mean overall recall rates for the three concurrent tasks. ANOVA yielded a significant main effect of VWM capacity (*F*(1, 38) = 56.87, *p* < 0.001, η^2^ = 0.60), indicating an overall higher recall rate in the high VWM capacity group (*M* = 78.44, *SD* = 20.76) than in the low VWM capacity group (*M* = 47.65, *SD* = 21.65). The main effect of concurrent task condition was also significant (*F*(2, 76) = 10.17, *p* < 0.001, η^2^ = 0.21). A comparison among pairs of means, using Bonferroni’s method, confirmed that the recall rate was higher in the control and simple tapping condition, which showed no difference between each other, than in the complex tapping condition (control condition, *M* = 71.72, *SD* = 24.35; simple tapping condition, *M* = 63.80, *SD* = 28.04; complex tapping condition, *M* = 53.62, *SD* = 23.36). A significant interaction effect was observed (*F*(2, 76) = 4.69, *p* = 0.012, η^2^ = 0.11). Multiple comparisons with Bonferroni’s method showed a significant difference in the high VWM capacity group with concurrent task conditions (*p* = 0.001), indicating that the recall rate was higher in the control (*M* = 84.38, *SD* = 16.66) and simple tapping conditions (*M* = 86.25, *SD* = 13.99), which showed no difference between each other, than in the complex tapping condition (*M* = 64.69, *SD* = 23.76). In addition, a significant difference was observed in the low VWM capacity group (*p* = 0.002), confirming that the recall rate was higher in the control condition (*M* = 59.06, *SD* = 24.54) than in the simple (*M* = 41.35, *SD* = 18.90) and complex tapping conditions (*M* = 42.55, *SD* = 17.26), which showed no difference between each other.

## Discussion

The results showed that the high and low VWM capacity groups were influenced differently by the two tapping tasks, i.e., with significantly higher recall rates in the control and simple tapping conditions than in complex tapping condition in the high VWM capacity group, while significantly higher recall rates were observed in the control condition than in the simple and complex tapping conditions in the low VWM capacity group. These results do not support our hypothesis, in which an interaction was not predicted. Combined with the observed main effect of VWM capacity and concurrent task condition, it was clarified that the degree of imagery representation formation differed depending on VWM capacity when imagery memory for spatial descriptions was required.

When drawing a map of spatial descriptions, more processing resources are distributed to the manipulation of imagery representations due to the non-retention of verbal representations. Listeners with high VWM capacity can construct verbal representations more efficiently, i.e., they utilize less processing resources so that more resources can be distributed to the construction and retention of imagery representations. In the simple tapping condition, though the simple physical movements required some processing resources of VSWM, the capacity gap could be filled rapidly using processing resources from VWM to maintain the imagery representations translated from verbal information. Therefore, there was no significant difference between the simple tapping and control condition. However, in the complex tapping condition, which requires not only physical movements but also attentional control through visual perception, processing resources have to be redistributed to prioritize continuous information processing of the input language. Consequently, the updating of prior formed imagery representations is interfered with. This means that more complete imagery representations could be retained in the control and simple tapping conditions, but only incomplete imagery representations and former verbal ones could be retained in the complex tapping condition. Conversely, listeners with low VWM capacity showed a decreased recall rate in both tapping conditions. Although the necessary processing resources for retaining verbal representations were reduced to some degree, the resources that could be distributed to VSWM were limited by the efficiency of constructing verbal representations. Combined with the observed main effect of VWM capacity, it can be inferred that in the tapping conditions, the updating of verbal representations and subsequent generation of imagery are disrupted by the reduction of processing resources due to spatial tapping. This causes more complete imagery representations to be retained in the control condition, but only incomplete imagery representations and former verbal ones are retained in the simple and complex tapping conditions.

From the results of Experiment 2, it is inferred that in the same time frame, the ability of the high VWM capacity group to construct verbal representations efficiently allows more processing resources to be distributed for the construction and maintenance of imagery representations, thereby resulting in more complete imagery representations of the spatial situation model than in those with low VWM capacity when imagery memory recall is required.

## General discussion

Several studies have revealed the significant roles of VWM and VSWM in spatial text processing in the general population (e.g., De Beni et al. 2005; Gyselinck et al. 2007, Pazzaglia et al. 2007). However, within this population, there are individual differences in VWM and VSWM capacity. To understand some of the variability in the performance of spatial text recall, Meneghetti et al. (2009) investigated individual differences in spatial ability. The present study investigated individual differences in VWM capacity as an independent variable, which reportedly to has a role in spatial text processing, to explore further how VSWM is involved in spatial text processing, in line with the WM model of Baddeley (2010).

In our experiments, concurrent tapping tasks were manipulated as a second independent variable. The scores of the free recall test (Experiment 1) and map drawing test (Experiment 2) were used as dependent variables. The results of Experiment 1 showed no differences in performance in the high VWM capacity group between the three tapping conditions; in the low VWM capacity group, a lower performance with both types of spatial tapping was observed in comparison to the control condition. The results of Experiment 2 showed that complex spatial tapping impaired performance in the high VWM capacity group in comparison to the simple spatial tapping and control conditions; in the low VWM capacity group, both spatial tapping tasks impaired performance. In addition, the participants with high VWM capacity demonstrated better performance than those in the low VWM capacity group. It could be concluded that the low VWM capacity group needs more verbal and visuospatial processing resources to construct a spatial situation model derived from verbal descriptions in comparison to the high VWM capacity group.

The differences in the interference effects between the high and low VWM capacity groups were consistent with those reported by Meneghetti et al. (2009), who suggested that high spatial ability can compensate for spatial WM interference; a rather low spatial ability leads to a requirement for a larger amount of verbal and spatial WM resources during spatial text processing. In line with this, the present study provided support for the WM model of Baddeley (2010), in which the subsystems function in parallel under the control of the central executive.

When spatial descriptions are included in listening comprehension, in which linguistic information is input sequentially along a time axis, VWM and VSWM function in parallel to construct a spatial situation model consisting of verbal and imagery representations. Verbal representations are constructed dependent on VWM and LTM in the episodic buffer. While these former verbal representations are retained temporarily in this buffer, the corresponding imagery representations are constructed in a VSWM and LTM-dependent manner. The imagery representations are then transferred to the episodic buffer and bound to the next incoming verbal representations.

The lack of an inference effect of both tapping tasks in the performance of the free recall test in the high VWM capacity group confirmed that increased VWM capacity enables the processing and retention of verbal information when verbal memory is required; rather, the impairment observed in the complex tapping task in the map drawing test confirms the need for VSWM function when imagery memory is required. In addition, the impairment caused by both tapping tasks in the free recall and map drawing tests in the low VWM capacity group reveals that the interference in the formation and binding of verbal and imagery representations is due to the need for more verbal resources. These observations demonstrate that the spatial situation model derived from spatial text is constructed through the same process, regardless of VWM capacity. However, the formation of VWM-dependent verbal representations and VSWM-dependent imagery representations and the binding of both in the episodic buffer, i.e., the abundance of modalities of the representations constituting the spatial situation model, varied between both groups with the requirements to the production of different representations. Accordingly, it can be concluded that the involvement of VSWM is constrained by the capacity of VWM in the construction of spatial situation models.

The present study characterized the construction of the spatial situation model in listening comprehension and addressed the involvement of VWM and VSWM in this process. However, a degree of caution is required in the interpretation of our findings in view of the small number of participants and the generalizability of the results. In addition, the interaction of high and low VSWM capacity with visuospatial ability and VSWM processing in spatial texts is an interesting issue that needs to be examined in future.

## Figures and Tables

**Fig. 4:**
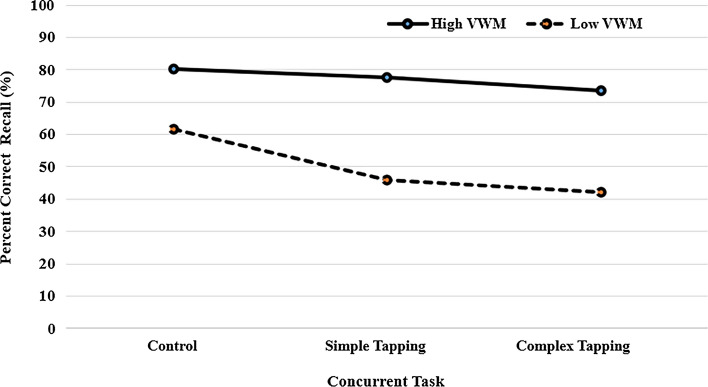
Verbal working memory capacity and concurrent task (Experiment 1)

**Fig. 5:**
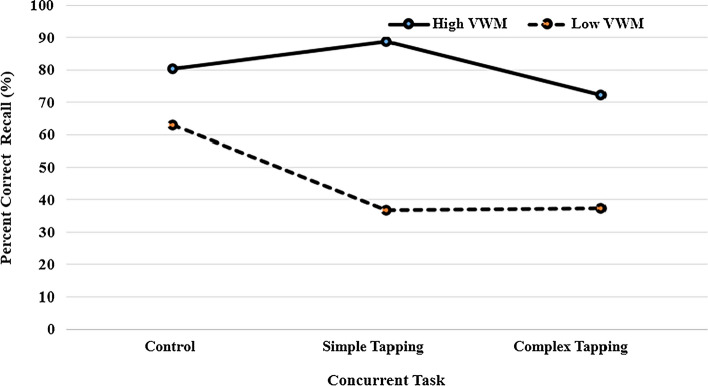
Verbal working memory capacity and concurrent task (Experiment 2)

## Appendix 1: Spatial texts used in Experiments

### Text 1

Xiaohua is taking a walk in the square. After entering the square, she walks straight for a little while and sees a fountain on her left. She turns right at the fountain and continues to walk a little further, where she sees a newly opened cafe. Next to the cafe is a flower store. When she reaches the cafe, she turns left and continues walking, with a botanical garden is on her right side.

### Text 2

Xiaoming is heading from Central Station to an interview with a company. Donghua Tower is in front of him after leaving the north exit of the station. He turns left as soon as leaves the station and continues walking for about eight minutes. Then he turns right at the third traffic light. After walking straight a little further, he sees a bus stop on his left side. The interviewing company is located on the 7th floor of the building behind the bus stop.

### Text 3

Xiaodong is participating in a marathon. The starting point of the course was in front of the university. He runs from the entrance of the university for a while and then turns left, a supermarket is on his right side. Next to the supermarket, there is a bookstore and a pharmacy. Turning right at the supermarket and continuing to run straight, he enters a pedestrian street. After coming out of the pedestrian street, he sees the city hall immediately in front of him.

## Appendix 2: Results of regression analysis of Experiment 1 and 2

### Experiment 1

Multiple linear regression analysis with interaction terms was performed to predict the recall rate in the free recall test based on VWM capacity and concurrent task. VWM capacity measured by LST was mean-centered and coded as “centered_VWM capacity.” Concurrent task was coded as “Dummy_simple tapping”: simple tapping = 1, ELSE = 0; “Dummy_complex tapping”: complex tapping = 1, ELSE = 0. The following interaction terms were created: “Interaction_simple × VWM” = Dummy_simple tapping × centered_VWM capacity; “Interaction_complex × VWM” = Dummy_complex tapping × centered_VWM capacity. A significant regression equation was found (*F*(5, 114) = 28.67, *p* < 0.001), with an R^2^ of 0.557. The participants’ predicted recall rate was equal to 0.709 + 0.129 (VWM capacity) − 0.090 (simple tapping) − 0.130 (complex tapping) + 0.085 (VWM capacity × simple tapping) + 0.086 (VWM capacity × complex tapping). When VWM capacity was low (= *M* − 1*SD*), the decline in the recall rate due to the concurrent task was significant. While when VWM capacity was high (= *M* + 1*SD*), the decline in the recall rate due to the concurrent task was much weaker (Fig. 4). VWM capacity, concurrent task, and interaction terms were all significant predictors of the recall rate in the free recall test (Table

**Table 3:** Results of multiple linear regression analysis (Experiment 1)

	Unstandardized Coefficients		Standardized Coefficients	t	Sig
Model	B	St. Error	Beta		
1. (Constant)	.709	.022		31.735	.000
Centered_VWM capacity	.189	.018	.678	10.664	.000
Dummy_simple tapping	− .090	.032	− .210	− 2.856	.005
Dummy_complex tapping	− .130	.032	− .302	− 4.122	.000
2. (Constant)	.709	.022		32.355	.000
Centered_VWM capacity	.129	.029	.463	4.437	.000
Dummy_simple tapping	− .090	.031	− .210	− 2.912	.004
Dummy_complex tapping	− .130	.031	− .302	− 4.202	.000
Interaction_simple × VWM	.085	.039	.188	2.190	.031
Interaction_complex × VWM	.086	.039	.190	2.213	.029

^3^).

### Experiment 2

Multiple linear regression analysis with interaction terms was performed to predict the recall rate in the map drawing test based on VWM capacity and concurrent task. The coding was the same as in Experiment 1. A significant regression equation was found (*F*(5, 114) = 22.66, *p* < 0.001), with an R^2^ of 0.498. The participants’ predicted recall rate was equal to 0.717 + 0.108 (VWM capacity) − 0.089 (simple tapping) − 0.169 (complex tapping) + 0.212 (VWM capacity × simple tapping) + 0.106 (VWM capacity × complex tapping). When VWM capacity was high (= *M* + 1*SD*), only the decline in the recall rate due to the complex task was significant. While when VWM capacity was low (= *M* − 1*SD*), the decline in the recall rate due to the concurrent task was significant (Fig. 5). VWM capacity, concurrent task, and interaction terms were all significant predictors of recall rate in the map drawing test (Table

**Table 4:** Results of multiple linear regression analysis (Experiment 2)

	Unstandardized Coefficients		Standardized Coefficients	t	Sig
Model	B	St. Error	Beta		
1. (Constant)	.717	.033		21.519	v
Centered_VWM capacity	.190	.024	.575	7.986	
Dummy_simple tapping	− .089	.047	− .156	− 1.879	.063
Dummy_complex tapping	− .169	.047	− .297	− 3.575	.001
2. (Constant)	.717	.031		23.379	.000
Centered_VWM capacity	.108	.029	.328	3.760	.000
Dummy_simple tapping	− .089	.043	− .156	− 2.041	.044
Dummy_complex tapping	− .169	.043	− .297	− 3.884	.000
Interaction_simple × VWM	.212	.046	.360	4.649	.000
Interaction_complex × VWM	.106	.046	.181	2.336	.021

^4^).

## Footnotes

1 Referring to Meneghetti et al. (2009), ANOVA was used. In addition, regression analysis with interaction terms was also conducted, and similar results were obtained. The results of the regression analysis of Experiment 1 and Experiment 2 are shown in Appendix 2.
